# Human entorhinal cortex electrical stimulation evoked short‐latency potentials in the broad neocortical regions: Evidence from cortico‐cortical evoked potential recordings

**DOI:** 10.1002/brb3.1366

**Published:** 2019-07-30

**Authors:** Hirofumi Takeyama, Riki Matsumoto, Kiyohide Usami, Takuro Nakae, Katsuya Kobayashi, Akihiro Shimotake, Takayuki Kikuchi, Kazumichi Yoshida, Takeharu Kunieda, Susumu Miyamoto, Ryosuke Takahashi, Akio Ikeda

**Affiliations:** ^1^ Department of Respiratory Care and Sleep Control Medicine Kyoto University Kyoto Japan; ^2^ Department of Neurology Kyoto University Kyoto Japan; ^3^ Division of Neurology Kobe University Graduate School of Medicine Kobe Japan; ^4^ Department of Epilepsy, Movement Disorders and Physiology Kyoto University Kyoto Japan; ^5^ Department of Neurosurgery Shiga Medical Center for Adults Moriyama Japan; ^6^ Department of Neurosurgery Kyoto University Kyoto Japan; ^7^ Department of Neurosurgery Ehime University Matsuyama Japan

**Keywords:** cortico‐cortical evoked potential, electrical stimulation, entorhinal cortex, hippocampus, memory

## Abstract

**Objective:**

We aimed at clarifying the clinical significance of the responses evoked by human entorhinal cortex (EC) electrical stimulation by means of cortico‐cortical evoked potentials (CCEPs).

**Methods:**

We enrolled nine patients with medically intractable medial temporal lobe epilepsy who underwent invasive presurgical evaluations with subdural or depth electrodes. Single‐pulse electrical stimulation was delivered to the EC and fusiform gyrus (FG), and their evoked potentials were compared. The correlation between the evoked potentials and Wechsler Memory Scale‐Revised (WMS‐R) score was analyzed to investigate whether memory circuit was involved in the generation of the evoked potentials.

**Results:**

In most electrodes placed on the neocortex, EC stimulation induced unique evoked potentials with positive polarity, termed as “widespread P1” (P1w). Compared with FG stimulation, P1w induced by EC stimulation were distinguished by their high occurrence rate, short peak latency (mean: 20.1 ms), small peak amplitude, and waveform uniformity among different recording sites. A stimulation of more posterior parts of the EC induced P1w with shorter latency and larger amplitude. P1w peak amplitude had a positive correlation (*r* = .69) with the visual memory score of the WMS‐R. In one patient, with depth electrode implanted into the hippocampus, the giant evoked potentials were recorded in the electrodes of the anterior hippocampus and EC near the stimulus site.

**Conclusions:**

The human EC electrical stimulation evoked the short‐latency potentials in the broad neocortical regions. The origin of P1w remains unclear, although the limited evidence suggests that P1w is the far‐field potential by the volume conduction of giant evoked potential from the EC itself and hippocampus. The significance of the present study is that those evoked potentials may be a potential biomarker of memory impairment in various neurological diseases, and we provided direct evidence for the functional subdivisions along the anterior–posterior axis in the human EC.

## 1 INTRODUCTION

The medial temporal lobe has been regarded as an important structure for episodic memory. The medial temporal lobe system for episodic memory is composed of the hippocampus and the perirhinal, entorhinal, and parahippocampal cortices. The entorhinal cortex (EC), which is located in the anterior parahippocampal gyrus, is considered the gateway between the hippocampus and neocortex (Squire & Wixed, 2011). Thus, it is important to know how the EC interacts with both the hippocampus and neocortex for a more detailed understanding of the episodic memory system.

Subdural electrode implantation is commonly performed for epileptic surgery. The two main purposes of electrode implantation are identification of the epileptogenic focus and functional brain mapping. We developed the cortico‐cortical evoked potential (CCEP) method as an invasive interventional approach to probe the causal influence, the so‐called “effective connectivity” (Matsumoto et al., 2004; Matsumoto & Kunieda, 2018). In this method, we apply single‐pulse electrical stimulations to specific cortical areas and record CCEPs time‐locked to the stimuli from remote cortices. This method can identify the effective connectivity between the stimulus site and remote neocortical region, which are presumably connected by a long association fiber tract (Yamao et al., 2014).

The CCEP study found in preliminary investigations that uniform sharply contoured positive potentials of short latency can be recorded from various neocortical regions after the stimulation of the medial temporal lobe. We termed such broadly recorded potentials as widespread P1 (P1w). P1w were observed in response to stimulation of the anterior parahippocampal gyrus, especially the EC. Such broad evoked response on various neocortical areas outside the limbic system has not been investigated yet, at least not in electrophysiological studies. Previous anatomical and electrophysiological studies investigating the connectivity between the EC and neocortex showed that connections are mainly found within the temporal lobe and that connections outside the temporal lobe are almost limited to limbic structures such as the cingulate gyrus and orbitofrontal area (Catenoix et al., 2005; Catenoix, Magnin, Mauguiere, & Ryvlin, 2011; Enatsu et al., 2015; Koubeissi, Kahriman, Syed, Miller, & Durand, 2013; Kubota et al., 2013; Lacruz, Seoane, Valentin, Selway, & Alarcon, 2007; Munoz & Insausti, 2005). However, in the recent human CCEP study using an effective connectivity model, it was revealed that the hippocampus could act as a signal amplifier of afferent information flow from the EC (Krieg et al., 2017). Therefore, EC electrical stimulation can have significant effects on broad neocortical regions outside the temporal lobe via such signal amplification of the hippocampus.

Along with CCEP, as part of the presurgical evaluations for epileptic surgery, patients in our institute usually undergo a series of neuropsychological batteries, including Wechsler Memory Scale‐Revised (WMS‐R). The WMS‐R has been used clinically as one of the standard batteries for the assessment of memory function. Medial temporal lobe epilepsy patients frequently experienced memory impairment, as reflected by the decrease in WMS‐R scores. The present study aimed to clarify the significance of P1w evoked by the human EC electrical stimulation. For this purpose, we investigated the characteristics of P1w, including its correlation with WMS‐R scores to verify whether P1w reflect the medial temporal lobe episodic memory circuit activity.

## 2 MATERIALS AND METHODS

### 2.1 Subjects

We recruited nine patients (five men and four women, 21–52 years old) with medically intractable medial temporal lobe epilepsy who underwent subdural electrode implantation into the EC (left: seven; right: two) from April 2011 to January 2015 in our hospital. The demographics of all patients are shown in Table 1. All patients provided written informed consent. The protocol was in accordance with the Declaration of Helsinki and was approved by the ethics committee of our institute (IRB #443).

**Table 1:** Patients' demographics and clinical information

Patient, age/gender, handedness	Electrode implanted side	Age of seizure onset	Seizure type	Ictal ECoG onset
A. 38F, R	L	29	Epigastric rising sensation → CPS	PHG
B. 29M, R&L	L	10	Aura (metamorphopsia, epigastric rising sensation) → CPS	PHG
C. 51M, R	L	43	CPS	mITG
D. 41F, R	L	19	Aura (nausea, feeling pale) → CPS	PHG
E. 22M, R	L	16	Nonspecific aura → CPS, GTCS	aMTG
F. 27F, R	R	16	Epigastric rising sensation → CPS	vAT
G. 28F, R	L	12	Precordial discomfort → CPS	PHG
H. 39M, L	R	12–15	Aura (epigastric rising sensation, fear) → CPS, GTCS	PHG
I. 21M, R	L	14	Aura (déjà vu, jamais vu, smell fit, epigastric discomfort) → CPS	vAT

Patient, age/gender, handedness	MRI	Pathology	WADA test (language, memory)
A. 38F, R	L hippocampal atrophy/sclerosis	HS^a^	L, L
B. 29M, R&L	L posterior temporal cortical atrophy	FCD IA HS^b^	B (L < R), B (L > R)
C. 51M, R	Left temporal cavernoma	AVM	L, B
D. 41F, R	L hippocampal atrophy/sclerosis, L parieto‐occipital perinatal infarction	FCD IA HS^b^	R, R
E. 22M, R	L basal frontal cortical dysplasia, L anterior temporal arachnoid cyst	FCD IA	L, L
F. 27F, R	R mesial temporal cyst	FCD IA	L, L
G. 28F, R	L hippocampal atrophy/sclerosis	HS^a^	L, B (L < R)
H. 39M, L	R hippocampal atrophy/sclerosis	FCD IA HS^b^	R, B (L < R)
I. 21M, R	L hippocampal atrophy/sclerosis	FCD IA HS^a^	L, B (L > R)

Patient, age/gender, handedness	WAIS‐R/III (VIQ, PIQ, TIQ)	WMS‐R (verbal, visual, general, attention, delayed recall)	WAB (AQ)
A. 38F, R	84, 97, 89	75, 111, 83, 62, 53	98.5
B. 29M, R&L	72, 78, 72	99, 92, 97, 87, 83	96
C. 51M, R	73, 97, 83	80, 101, 85, 101, 91	89.6
D. 41F, R	72, 83, 75	83, 111, 89, 94, 82	97.3
E. 22M, R	70, 78, 69	99, 64, 87, 91, 82	95.6
F. 27F, R	106, 102, 105	112, 114, 114, 81, 100	99.6
G. 28F, R	62, 80, 67	64, 94, 68, 79, 79	95.8
H. 39M, L	93, 105, 98	74, 94, 77, 110, 96	99
I. 21M, R	86, 79, 81	55, 79, 53, 90, 54	97.4

Abbreviations: aMTG, anterior part of the middle temporal gyrus; AQ, Aphasia Quotient; AVM, arteriovenous malformation; B, bilateral; CPS, complex partial seizure; ECoG, electrocorticogram; FCD, focal cortical dysplasia (Palmini classification); GTCS, generalized tonic clonic seizure; HS, hippocampal sclerosis; L, left; mITG, middle part of inferior temporal gyrus; PHG, parahippocampal gyrus; PIQ, Performance IQ; R, right; TIQ, Total IQ; vAT, ventral anterior temporal; VIQ, Visual IQ; WAB, Western Aphasia Battery; WAIS, Wechsler Adult Intelligence Scale; WMS‐R, Wechsler Memory Scale‐Revised.

a Diagnosed by clinical findings.

b Dual pathology.

### 2.2 Electrode placement

**Figure 1:**
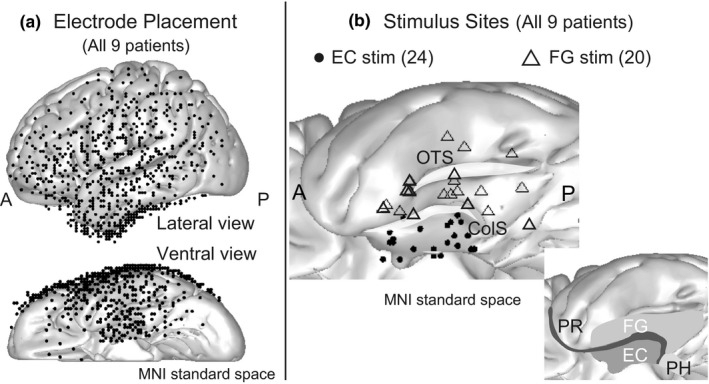
Electrode placement and stimulus site. (a) Locations of the electrodes in all patients are shown as black dots in the MNI standard space (upper, lateral convexity; lower, basal area). All stimulus and recording electrodes are projected onto the left hemisphere for display purposes. (b) The left upper figure shows the stimulus sites. The midpoints of the stimulus electrode pairs are plotted as black dots (EC stimulation) or triangles (FG stimulation). The right lower panel illustrates the anatomical relationship among the subregions of the medial temporal lobe (revised from figure 2 in Squire & Wixed, 2011). ColS, collateral sulcus; EC, entorhinal cortex; FG, fusiform gyrus; OTS, occipito‐temporal sulcus; PH, parahippocampal cortex; PR, perirhinal cortex

Electrode locations for all patients are shown in Figure 1a. The electrodes were implanted in medial and lateral temporal, orbitofrontal, lateral frontal, and lateral parietal areas. After excluding the electrodes inappropriate for the analysis due to bad recording condition because of the disconnection of the electrode wire or floating of the electrode from the brain surface, the number of total electrodes and electrodes per patient was 792 and in the range of 63–107 [88 ± 14.3, mean ± standard deviation (*SD*)], respectively.

### 2.3 Stimulus sites

Stimulus sites for all patients are summarized in Figure 1b, which shows the stimulus sites as the midpoints of electrode pairs in MNI standard space. The total numbers of stimulus sites in the EC and the fusiform gyrus (FG) were 24 and 20, respectively. The number of stimulus sites per patient varied between one and five (2.7 ± 1.4) for the EC and between one and five (2.2 ± 1.2) for the FG. Current intensities of the stimuli ranged from 6 to 12 mA (9.4 ± 1.6 mA).

### 2.4 Cortico‐cortical evoked potentials

The methodology of CCEPs has been previously described in detail (Matsumoto et al., 2004, 2007, 2012). Using a constant‐current stimulator (MS‐120B/MEE‐1232; Nihon Kohden), direct bipolar electrical stimulation was applied to a pair of adjacent subdural electrodes (platinum; recording surface diameter, 2.3 mm; interelectrode distance, 1 cm; AD‐TECH, WI). Single‐pulse electrical stimuli (square‐wave pulse, 0.3 ms duration) were applied at 1 Hz with alternating polarity. A total of 60 (100 for patient E) single pulses were delivered to each stimulus site.

The aim of CCEPs was to identify functional and seizure networks by stimulating the majority of implanted electrodes for research and clinical purposes (Kobayashi et al., 2015; Matsumoto, Kunieda, & Nair, 2017). We used the highest current intensity at which (a) the patient did not notice the stimulation and no apparent symptoms were evoked, (b) adjacent electrodes did not show excessive artifacts interfering with the recording, and (c) no afterdischarges were detected. Current intensity was adjusted by increments of 1 or 2 mA reaching a maximum current between 6 and 12 mA. In some stimulus pairs, we could not reach a maximum current intensity (12 mA) due to symptoms such as pain, excessive artifacts in adjacent electrodes, or afterdischarges. Patients were awake during stimulation, except during the sleep investigations described below. Electrocorticogram (ECoG) recordings were referenced to a scalp electrode on the skin over the mastoid process contralateral to the side of electrode implantation. Recordings were sampled at 1,000 Hz with a band‐pass filter of 0.08–300 Hz (patient E: sampling 2,000 Hz, band‐pass filter 0.08–600 Hz). ECoG recordings were averaged offline time‐locked to the electrical stimuli (analysis window: −100 to +900 ms; baseline window: −100 to −5 ms; time 0: onset of stimuli).

### 2.5 Definition of and inclusion criteria for P1w potentials

**Figure 2:**
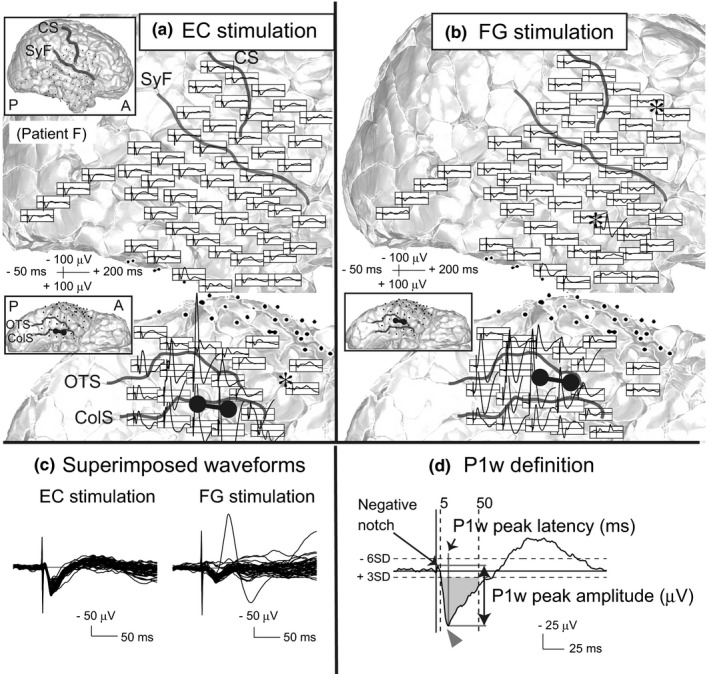
Representative cortico‐cortical evoked potential (CCEP) waveforms and P1w definition. (a,b) CCEPs evoked by EC (a) and FG (b) stimulation in a representative case (patient F). CCEPs (black waveforms) are plotted on a 3D MRI. The left upper panel in each brain map shows the whole 3D MRI to facilitate an understanding of the electrode configuration. The vertical line corresponds to the time of stimulation. In the 3D MRI, each stimulus site is illustrated by a pair of interconnected black electrodes. Electrodes that recorded local maxima of remote isolated fields are marked by asterisks (*). (c) Superimposed CCEP waveforms of lateral surface electrodes. Note that CCEPs by EC stimulation are more uniform than those by FG stimulation. (d) P1w definition. Representative CCEP evoked by EC stimulation in patient D (recorded from the primary somatosensory cortex). The arrowheads indicate P1w peaks. The area shaded in gray indicates the inclusion criteria for P1w as defined in Section 2. Peak latency must be between +5 and +50 ms, and peak amplitude must exceed three *SD* of baseline activity. ColS, collateral sulcus; CS, central sulcus; EC, entorhinal cortex; FG, fusiform gyrus; OTS, occipito‐temporal sulcus; SyF, Sylvian fissure

As stated in the Section 1, we considered the short‐latency potentials of positive polarity as the most striking component of CCEP waveforms in response to EC stimulation, or FG stimulation (less prominent; Figure 2a–c). As stated in the Section 1, we called these short‐latency potentials of positive polarity as P1w. The inclusion criteria for P1w were defined as follows (Figure 2d):

1. P1w is the earliest deflection of positive polarity.
2. The peak amplitude of P1w is more than three times larger than the *SD* within the baseline window.
3. The peak latency of P1w is in the range from +5 to +50 ms after the stimulation.
4. P1w is not followed by a typical or late N1 potential (described below).

We defined only potentials satisfying all four criteria as P1w. Here, N1 is the earliest deflection of negative polarity. In typical CCEP recordings, we usually observe local responses near the stimulus site and discrete remote responses. Both nearby and remote CCEP waveforms typically show N1 and N2 potentials. Remote responses are usually spatiotemporally separated from nearby responses, and those responses form one or more electrical fields (herein defined as “remote isolated field”) on the area far from the stimulus site (see also Figure 2a,b). Typically, CCEP studies consider only N1 with a peak latency between 5 and 50 ms after the stimulation (“typical N1”; Matsumoto et al., 2017, 2007, 2004; Figure S1A). However, in our preliminary analysis, the peak latency of N1 evoked by either the EC or FG stimulation occasionally exceeded 50 ms, especially in electrodes located in lateral temporal and parietal areas (“late N1”; Figure S1B).

Based on these observations, we defined N1 as the earliest deflection of negative polarity with an amplitude exceeding six times the *SD* within the baseline window (Keller et al., 2014). In addition, we defined that it is between +5 and +50 ms when the upslope of N1 crosses the six *SD* threshold for the first time (Figure 2d, Figure S1). Therefore, after excluding the data obtained from the electrodes that recorded such N1 potentials for further analysis, it is always after +50 ms when the upslope of the blunted negativity following P1w happens to cross the six *SD* threshold for the first time. We think this N1 definition can differentiate typical and late N1 of CCEP waveforms in remote isolated fields from the blunted negativity following P1w potentials and eventually help in extracting the essential features of P1w potentials.

The caveat is that in our previous CCEP study of the dorsal language network, we called the preceding trough of N1 with a positive polarity as P1, which is regarded as the first component of a direct white matter pathway (Yamao et al., 2014). That P1 is different from P1w potentials investigated in this study.

### 2.6 Representative indices of P1w potentials

We analyzed peak latencies and peak amplitudes along with their presence as representative indices of P1w and compared them for EC and FG stimulation. We also investigated whether the locations of stimulus sites within the EC had an influence on these parameters.

Recording electrodes located within 25 mm from a stimulation electrode were excluded from the analysis to eliminate local effects, presumably through short U‐fibers (Keller et al., 2011). Electrodes in bad recording conditions, for example, disconnected or floating electrodes, were also excluded. For the detection of P1w peaks, we used custom MATLAB scripts (developed by M.M.).

#### 2.6.1 P1w peak amplitude (μV)

In a preliminary analysis of P1w evoked by EC stimulation, stimulus artifacts were almost always followed by a small notch of negative polarity. We defined the P1w peak amplitude as the voltage difference between the peaks of the poststimulus negative notch and the P1w potential (Figure 2d). The timing of the poststimulus negative notch was determined for each stimulus site by visual inspection. When P1w had multiple positive peaks, the largest peak was used.

#### 2.6.2 P1w peak latency (ms)

The peak latency of P1w was defined as the time between stimulation and P1w peak (Figure 2d).

#### 2.6.3 P1w presence

We also analyzed how many electrodes per stimulus site recorded a P1w potential. We assigned each recording electrode to a nominal “P1w presence” scale, with P1w being either present or absent.

#### 2.6.4 P1w presence ratio (%)

This parameter was defined as the percentage of electrodes that recorded a P1w potential. Subdural electrodes within 25 mm from the stimulus site were excluded.

#### 2.6.5 Contour map of P1w peak latency and amplitude

To analyze the effects of stimulus locations within the EC on P1w peak latencies and amplitudes, we generated 3D contour map in MNI standard space for both indices on the cortical surface of the EC, which reflected P1w peak latencies and amplitudes. The midpoint of each electrode pair was employed to represent the coordinates of this stimulus site. P1w indices were averaged among P1w‐present electrodes for each stimulus site. Then, these averaged values were plotted at the midpoint of each stimulation electrode pair. We also correlated P1w peak indices, that is, latency and amplitude, with the locations of EC stimulus sites. The correlation was investigated along the *X*‐ and *Y*‐coordinates of the MNI standard space, representing the medial–lateral and anterior–posterior axes, respectively.

### 2.7 Anatomical definition of the EC and FG stimulus sites

Based on the method used by Franko, Insausti, Perula, Insausti, and Chavoix (2014), the anterior and posterior borders of the EC were defined according to positions of key landmarks (e.g., limen insulae and the end of the gyrus intralimbicus) in the preoperative brain MRI. An MRI taken during implantation was coregistered to the preoperative MRI to determine whether an electrode was located within the border of the EC in the anterior–posterior axis. The lateral border of the EC was defined as the collateral sulcus (ColS). The ColS was also identified in MRIs taken during implantation. The inclusion criteria for EC stimulation were defined as follows:

1. Anterior–posterior axis: Either electrode of the stimulus pair must be located in the EC.
2. Medial–lateral axis: Both electrodes of the stimulus pair must be located in the parahippocampal gyrus medial to the ColS.

To clarify the features of P1, we used the fusiform gyrus as a control stimulus site. The inclusion criteria for FG stimulation were defined as follows:

1. Anterior–posterior axis: Both electrodes of the stimulus pair must be located in the FG. The ColS and occipito‐temporal sulcus represent the medial and lateral boundaries of the FG, respectively.

### 2.8 Differences in functional connectivity between the EC and FG stimulation: the number of remote isolated fields

As previously described, in typical CCEP recordings, local responses near the stimulus site and discrete remote responses are usually observed. Remote responses are usually spatiotemporally separated from nearby responses, and those responses form one or more electrical fields (herein defined as “remote isolated field”) on the area far from the stimulus site. A recent intraoperative cortical and subcortical stimulation study indicates that the N1 component of such remote isolated field potentials is generated by the long association fiber tract (Yamao et al., 2014). We hypothesized that a comparison of remote isolated fields evoked by EC or FG stimulation elucidates the differences between their functional connectivity in addition to P1w analysis. We defined the local maximum of a remote isolated field as the electrode that showed the largest N1 amplitude of those electrodes within a remote isolated field and counted the number of the electrodes with the local maxima of the remote isolated field per stimulus site (see also Figure 2a,b; electrodes that recorded local maxima of remote isolated fields are marked by asterisks [*] in those figures). All remote isolated fields were evaluated by two authors independently (H.T. and R.M.). If the evaluation differed between the reviewers, the remote isolated field was defined by agreement.

### 2.9 Anatomical localization of electrodes in individual and standard spaces

The methods of standard electrode placement and coregistration to the MNI standard space are described in detail elsewhere (Matsumoto et al., 2004, 2011). In short, magnetization‐prepared rapid gradient‐echo (MP‐RAGE) sequences as anatomical T1‐weighted volume data were obtained before and after electrode implantation. We determined the electrode coordinates in the image taken after implantation by the hypointense signal caused by the electrode's platinum alloy properties. Next, we coregistered these coordinates for each patient nonlinearly to the scan image obtained before implantation and mapped this to the MNI standard space (ICBM‐152) using FNIRT (www.fmrib.ox.ac.uk/fsl/fnirt). Pictures of electrodes in the right hemisphere (patients F and H) are shown in figures horizontally flipped for display purposes.

### 2.10 Cortico‐cortical evoked potentials during sleep

To exclude the possibility that P1w potentials were stimulus artifacts, we also investigated the state‐dependent changes of P1w indices (peak latency, peak amplitude, presence ratio) at different sleep stages (awake [W], light sleep [L], slow‐wave sleep [SWS], REM sleep [REM]). We used the same criteria mentioned above to define EC stimulation. Stimulus electrode pair, current intensity, and frequency were kept identical in each patient throughout all stages of sleep. Sleep stages were determined offline by scalp electroencephalography (Usami et al., 2015).

We evoked sleep CCEPs in three patients (patients F, H, and I). Stimulation parameters (stimulus intensity, frequency) varied among these patients (patient F, 6 mA, 1 Hz; patient H, 6 mA, 0.2 Hz; patient I, 5 mA, 0.5 Hz). Stimulation and recording conditions were otherwise the same for CCEPs in awake and sleeping patients except for a higher sampling rate during sleep (2,000 Hz). The number of recording electrodes had to be reduced for sleep CCEP recordings due to EEG amplifier specifications (fewer electrode recording with a higher sampling rate; patient F, 32; patient H, 42; patient I, 44). In general, 40–80 electrical pulses were delivered at each sleep stage.

### 2.11 Correlation between P1w peak amplitude and neuropsychological scores

As part of the presurgical evaluations, all patients underwent a series of neuropsychological batteries, including WMS‐R. To investigate whether P1w reflect the activity of the circuit important for episodic memory, we analyzed the correlation between WMS‐R memory quotient (MQ; verbal, visual, delayed recall) and P1w peak amplitude of the EC stimulation. For this analysis, we averaged the P1w peak amplitude across all recording electrodes for each stimulus site. We then grand‐averaged the P1w peak amplitude for EC and FG stimulation separately in each individual patient, when the stimulation is delivered to more than one electrode pair in each region. Recently, Lee, Ryu, Lee, Kim, and Lee (2016) showed in human fMRI study that the efficient retrieval of object–place paired associate memory was correlated with the BOLD response of the left hippocampus, whereas the efficient retrieval of relatively pure spatial memory was correlated with the right hippocampal BOLD response, suggesting that the left and right hippocampus process qualitatively different information for remembering episodic events in space. Thus, we expect that both the left and right hippocampus may have an important role in episodic memory function. According to such expectation, we put the data of all nine patients together for statistical analysis regardless of the side of the electrode implantation in order to improve the sensitivity.

### 2.12 Data analysis

CCEP analysis, including auto‐detection of P1 peaks, was performed by MATLAB (R2013a) and custom scripts developed by M.M. Statistical analyses were performed with JMP Pro 11. 3D projection of P1w latency and amplitude values onto the brain surface in the MNI standard space was achieved by custom MATLAB scripts (developed by T.N.).

## 3 RESULTS

### 3.1 Comparison of P1w indices between EC and FG stimulation

**Figure 3:**
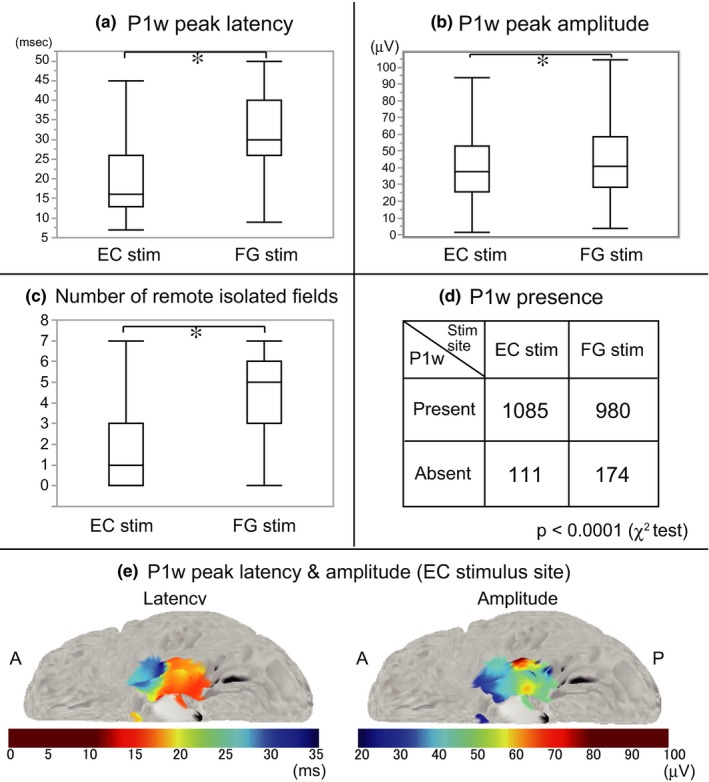
Comparison of P1w indices and the number of remote isolated fields for EC and FG stimulation, and contour map of P1w indices in MNI standard space. (a–c) The box plots show the results of P1w peak latency (a), P1w peak amplitude (b), and the number of remote isolated fields (c). *p < .05, EC versus FG stimulation, Mann–Whitney test. (d) P1w presence after EC and FG stimulation. The number of P1w‐present electrodes was significantly larger in EC compared to FG stimulation (*p* < .0001, *χ* ^2^ test). (e) Contour map of P1w peak latency and amplitude averaged across all patients. P1w indices are plotted on the left EC stimulus sites for display purposes. The upper and lower limits of the color scale are defined according to the maximum and minimum values of P1w indices in box plots of Figure 3a,b

To investigate whether P1w indices were different between EC and FG stimulation, we compared the P1w indices of all recording electrodes from all patients (EC stim. 1085, FG stim. 980 [electrodes]; Figure 3a). Differences between EC and FG stimulation reached significant levels for all P1w indices—P1w peak latency (Figure 3a; EC stim., 20.1 ± 10.0; FG stim., 32.3 ± 9.0 [ms]; *p* < .0001, Mann–Whitney test), P1w peak amplitude (Figure 3b; EC stim., 43.0 ± 26.0; FG stim., 50.0 ± 38.4 [μV]; *p* < .0001, Mann–Whitney test), and P1w presence ratio (Figure 3d; EC stim., P1w present 1085: P1w absent 111 [electrodes], P1w presence rate 90.7%; FG stim., P1w present 980: P1w absent 174 [electrodes], P1w presence rate 84.9%; *p* < .0001, *χ* ^2^ test). The time of the negative notch ranged between +2 and +6 ms (3.7 ± 0.8 ms).

The contour map of P1w peak latencies and amplitudes at the stimulus sites in the EC (Figure 3e) shows their gradation along the anterior–posterior axis, indicating that a stimulation of the more posterior part of the EC tended to evoke P1w with shorter peak latency and larger peak amplitude. Regression line analysis along the anterior–posterior axis (*Y*‐coordinate of the MNI space) revealed a moderate‐to‐high positive correlation between P1w peak latencies and the locations of the stimulus sites (*r* = .53, *p* < .0001), and a mild negative correlation between P1w peak amplitudes and the locations of the stimulus sites (*r* = −.33, *p* < .0001).

In summary, EC in comparison with FG stimulation evoked P1w in a higher number of electrodes, and their peaks have significantly shorter latencies and smaller amplitudes. Comparing within the EC group, a more posterior EC stimulation evoked P1w with shorter peak latency and larger peak amplitude.

### 3.2 Number of remote isolated fields

For conventional remote CCEP responses, the number of remote isolated fields per stimulus site was significantly smaller in EC stimulation compared to FG stimulation (Figure 3c; EC stim., 1.8 ± 1.9 [*n* = 24]; FG stim., 4.6 ± 2.1 [*n* = 20]; *p* = .0002, Mann–Whitney test). In EC stimulation, the local maxima of remote isolated fields were located mainly in the temporal lobe and the orbitofrontal area, which is consistent with previous human CCEP and monkey tracer studies (Munoz & Insausti, 2005). On the other hand, in FG stimulation, the local maxima of remote isolated fields were located not only in these areas but also in the lateral prefrontal and lateral parietal areas (see Figure S2).

### 3.3 Sleep CCEPs

**Figure 4:**
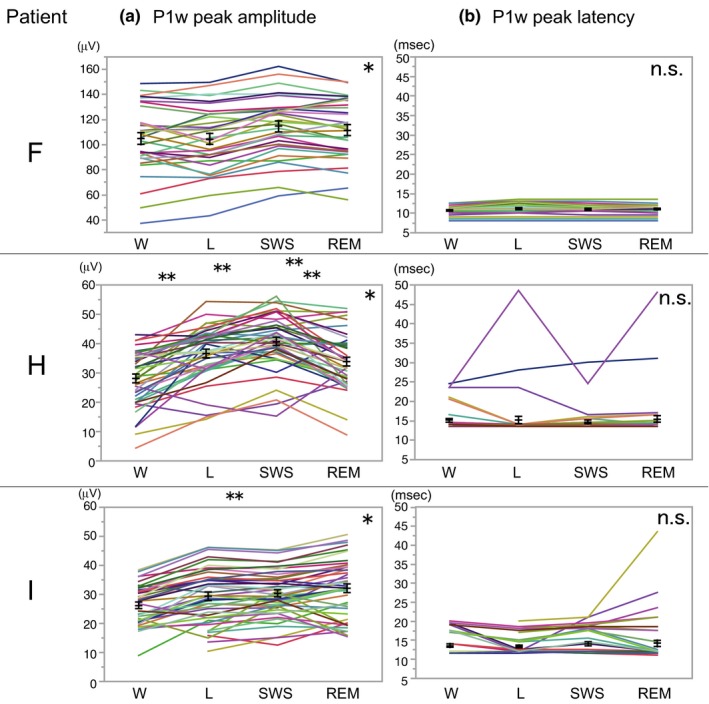
Cortico‐cortical evoked potential modulation during sleep. (a) P1w peak amplitude variations at different sleep stages in patients F, H, and I. *p < .0001, repeated‐measure ANOVA for each patient; **p < .05, Tukey–Kramer HSD post hoc analysis. (b) P1w peak latency did not change at different sleep stages. L, light sleep; REM, REM sleep; SWS, slow‐wave sleep; W, awake. Black lines: mean ± standard error

The P1w presence ratios were 100% at all sleep stages in all patients except for the awake (W) period of patient I with 91.9% (Figure 4). Thus, we did not analyze P1w presence ratios further in the sleep CCEP study because the statistical difference of P1w presence ratio among sleep stages is supposed to be absent.

P1w peak amplitudes for each recording electrode changed with sleep stages in all three patients (patient F, *F* (3, 93) = 24.4, *p* < .0001; patient H, *F* (3, 123) = 40.7, *p* < .0001; patient I, *F* (3, 123) = 47.5, *p* < .0001; repeated‐measure ANOVA for each patient; Figure 4a). A post hoc analysis using Tukey–Kramer HSD revealed significant differences for W‐L (*p* = .0003), W‐SWS (*p* < .0001), W‐REM (*p* = .0269), and SWS‐REM (*p* = .0056) in patient H, and for W‐REM (*p* = .0314) in patient I. Thus, in two out of three patients, P1w peak amplitudes increased significantly in the REM state compared to the awake state. On the other hand, P1w peak latencies did not change significantly at different sleep stages (Figure 4b).

### 3.4 Correlation between P1w peak amplitude and WMS‐R scores

**Figure 5:**
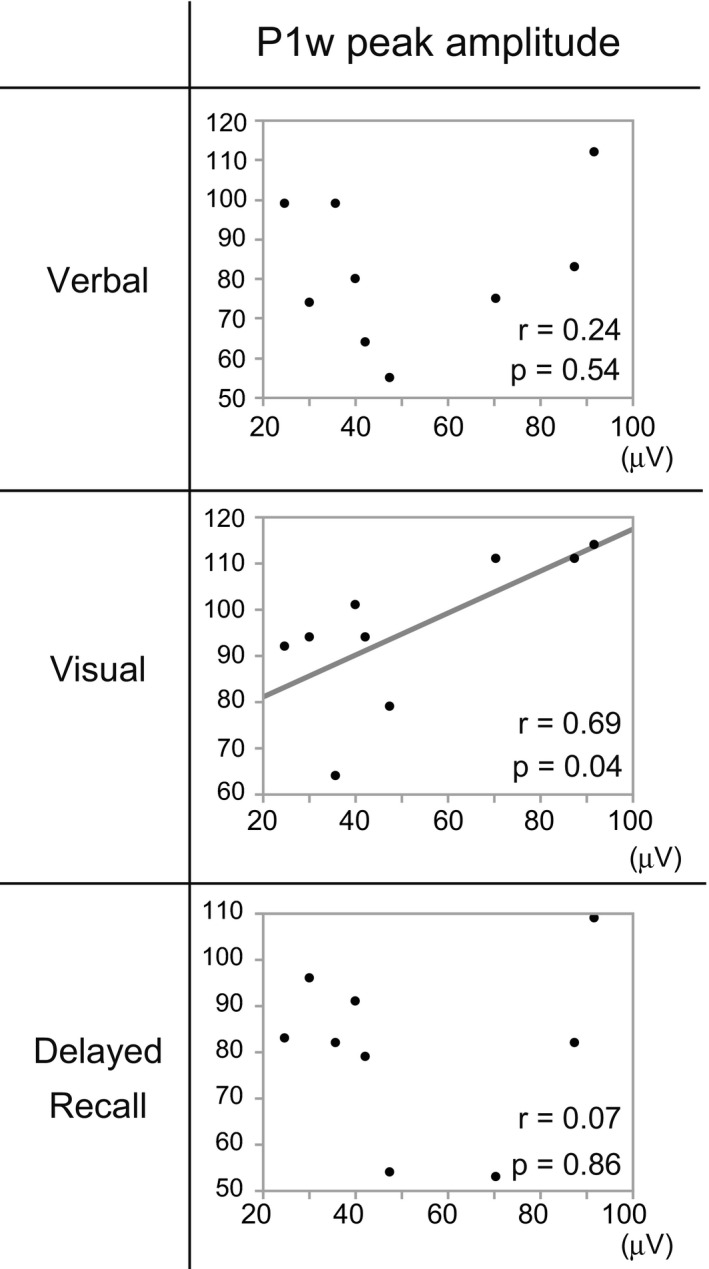
P1w peak amplitude and WMS‐R MQ scores. Linear regression analysis between P1w peak amplitude and WMS‐R. The horizontal axis denotes the value of P1w peak amplitude and the vertical axis MQ scores. Moderate‐to‐high significant correlations are observed between P1w peak amplitude and visual MQ of WMS‐R (*r* = .69)

WMS‐R was performed as part of the presurgical evaluations in all patients (Figure 5). We analyzed the correlation between WMS‐R MQ scores and P1w peak amplitude of the EC stimulation (Figure 5). Linear regression analysis revealed a moderate‐to‐high positive correlation between P1w peak amplitude and visual MQ of WMS‐R (*r* = .69). Only visual MQ had a statistically significant correlation with P1w peak amplitude (P1 peak amplitude–visual MQ, *p* = .04, uncorrected for multiple comparisons); however, the level of the correlation did not survive a correction of multiple comparisons (Tukey–Kramer HSD) probably due to the limited number of subjects (*n* = 9). In addition, we analyzed the correlation between WMS‐R MQ scores and P1w peak amplitude in FG stimulation. However, there were only mild, nonsignificant correlations (P1w peak amplitude–visual MQ, *r* = .34, *p* = .37).

### 3.5 Giant evoked potentials in the hippocampus and EC near the stimulus site

**Figure 6:**
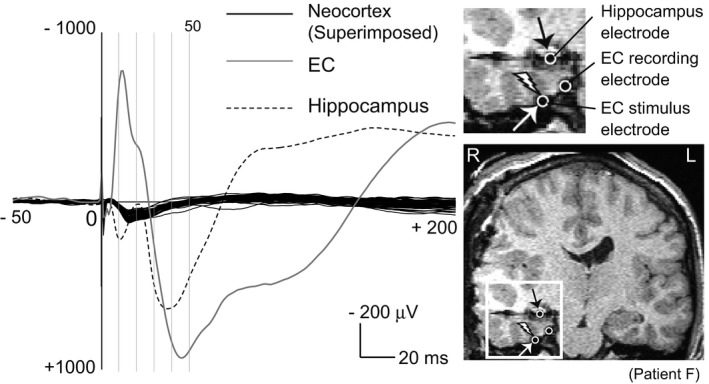
Large evoked potentials in the EC and hippocampus electrode near the stimulus site. The right lower figure shows coronal section of an MRI taken after electrode implantation in patient F. The area enclosed by the white square is enlarged in the right upper figure. Electrodes are hypointense signals due to the property of the platinum alloy. The left figure shows the evoked potentials by the EC stimulation. White open circle with a thunder symbol indicates location of stimulus electrode for EC stimulation. For EC stimulation, only one pair of stimulating electrodes is visible in the coronal section. The black solid lines are the superimposed cortico‐cortical evoked potential (CCEP) waveforms. Only CCEP waveforms recorded from the lateral brain surface are superimposed. The gray solid line and black dotted line are the evoked response recorded in the EC recording electrode (white open circle) and the hippocampus electrode (white open circle with black arrow), respectively

We found that EC stimulation evoked local responses with very high amplitude, up to a few millivolts (Figure 6). In one patient, patient F, evoked responses with high amplitude up to one millivolt were also recorded at the subdural electrode in the EC near the stimulus site, and at the depth electrode in the anterior hippocampus (Figure 6). In this patient, the earliest peak latencies of such giant evoked potentials preceded the peak latency of P1w by a few milliseconds.

## 4 DISCUSSION

### 4.1 Identified features of P1w induced by EC stimulation

EC stimulation induced in the majority of implanted neocortical electrodes unique evoked potentials with positive polarity, termed as P1w. The prominent features of P1w in EC stimulation were their waveform similarity, high occurrence rate, short peak latency, and small peak amplitude, demonstrated by a comparison of P1w indices during EC and FG stimulation (Figure 3a,b,d).

We consider the two main reasons for the lack of reports about P1w in previous CCEP studies. First, those CCEP studies mainly focused on spatially localized discrete responses with a typical pattern of negative polarities (N1 and N2; Keller et al., 2014; Swann et al., 2012; Yamao et al., 2014); these features are in contrast to those of P1w, which include positive polarity or nonlocalized spatial distribution. Second, the CCEP studies of the limbic system usually focused on the responses recorded in the depth electrodes, which cover the much narrower neocortical regions than the subdural electrodes do.

### 4.2 Origin of P1w

The duration of P1w was so long that a stimulus artifact seems unlikely as the origin of P1w. Sleep CCEPs demonstrated the modulation of P1w peak amplitudes by sleep stages (Figure 4) similar to conventional N1/N2 amplitudes (Usami et al., 2015). The absence of discrete evoked potentials in the reference electrode, which was located on the contralateral mastoid process, excluded the possibility that P1w were merely a consequence of a reference electrode activation (Figure S3). These observations led us to regard P1w as the real signal derived from the brain.

The waveform similarities of P1w across recording sites led us to consider the possibility that P1w is equivalent to the far‐field potential by the volume conduction. In addition, in three patients, in whom we added the scalp electrode for the sleep staging in sleep CCEP, the P1w potentials were recorded in not only the intracranial subdural electrodes but also in the scalp electrodes (Figure S4), supporting the hypothesis that the origin of P1w is the far‐field potential. In the electrophysiological research field, when the similar waveforms are recorded regardless of the locations of the recording electrodes in the monopolar montage, such responses are typically regarded as far‐field potentials. The concept of far‐field potential was firstly introduced in the auditory brainstem response study (Jewett & Williston, 1971), followed by the somatosensory evoked potential study (Cracco, 1972). Cracco showed that the evoked potentials in the deep subcortical structures could be recorded as far‐field potential in the broad areas on the scalp by the volume conduction. Previous studies of rodent models showed that the volume‐conducted currents from the hippocampus and EC contributed to the local field potentials recorded in the neocortex (Bland & Whishaw, 1976; Gerbrandt, Lawrence, Eckardt, & Lloyd, 1978; Sirota et al., 2008). We found that EC stimulation evoked giant local responses in the EC near the stimulus site and the hippocampus and that their earliest peak latencies preceded the peak latency of P1w by a few milliseconds (Figure 6). In the recent human CCEP study using an effective connectivity model, Krieg et al. (2017) revealed that the hippocampus could act as a signal amplifier of afferent information flow from the EC. We hypothesize that the signal amplification property of the hippocampus could augment the effects of the EC electrical stimulation and serve the generation of the giant evoked potentials in the EC and hippocampus, resulting in the volume‐conducted currents to broad neocortical regions and the observation of P1w potentials. Moreover, such amplification effect of the EC stimulation by the hippocampus would reflect some aspects of the memory network dynamics in the medial temporal lobe, suggested by the significant correlation between P1w peak amplitude and WMS‐R score (visual MQ) as we found in this study.

We found that the values of P1w peak latencies in EC stimulation were surprisingly consistent despite variable distances between the EC and recording sites (Figure 2a,c). However, in a typical CCEP, the N1 onset and peak latency vary according to the fiber length between the stimulus and recording site (Matsumoto et al., 2012). Thus, direct cortico‐cortical projections are unlikely to be the source of P1w.

If P1w were derived from the indirect cortico‐subcortical–cortical projections, the hippocampus and the thalamus are the two candidates for a subcortical relay point. The mean peak latency of P1w was 20.1 ms. Yeckel and Berger (1990) showed in a rabbit study that the electrical stimulation of EC afferents to the hippocampus caused multisynaptic excitation of CA1 pyramidal neurons via the trisynaptic pathway with latencies of 16–21 ms. Thus, from the point of conduction velocity, the EC–hippocampus–thalamo‐cortical projection is unlikely to be the origin of P1w.

In summary, we regard the far‐field potential from medial temporal structures as the most likely origin of P1w. However, we provided only indirect supportive evidence for this hypothesis. We hope a further study to clarify whether P1w is a far‐field potential or not.

### 4.3 P1w evoked by FG stimulation

We observed that the stimulation of the FG also elicited P1w. The FG is adjacent to the perirhinal cortex. The perirhinal cortex is located within the collateral sulcus (Figure 1b, right lower panel) and has strong connections with both the hippocampus and EC. According to the electrical density simulation study (Nathan, Sinha, Gordon, Lesser, & Thakor, 1993), the relatively high stimulation intensity we used for the fusiform gyrus might have activated the perirhinal cortex at the superficial part of the collateral sulcus. Then, the stimulation of the perirhinal cortex would have activated both the hippocampus and EC and resulted in the generation of P1w.

### 4.4 Neuroscientific implications: memory and P1w

We uncovered that the peak amplitudes of P1w were smaller in patients with a lower visual MQ score (Figure 5). That correlation between P1w peak amplitude and WMS‐R score, which may be explained by the axonal loss in the memory circuit from the EC to the hippocampus due to intractable epilepsy, supports the idea that P1w reflects the activity of the medial temporal lobe memory circuit. The changes of P1w peak amplitudes at different sleep stages (Figure 4) may reflect the sleep modulation on the activity of the memory circuit as the underlying mechanism for efficient memory consolidation during sleep.

Typically, verbal memory is considered as predominantly processed in the left hemisphere, which is in most cases dominant for language function, and visual memory in the right hemisphere. However, as Table 1 shows, moderate‐to‐severe visual memory deficit was only observed in the patients with left side lesion (patient E and I), although in these patients the dominant hemisphere for language function was the left side. Those clinical findings would explain the curious finding that the peak amplitude of P1w had good concordance to the visual memory scores despite that many of the patients were examined with a left hemisphere coverage. These clinical findings also suggest that the lateralization of visual and verbal memory function may depend on the patients.

However, the correlation of P1w peak amplitude with visual memory MQ did not survive a correction of multiple comparisons (Tukey–Kramer HSD). Thus, further investigation is needed to confirm whether P1w potentials really reflect the activity of the memory circuit.

We clarified that a stimulation of more posterior parts of the EC induced P1w with shorter peak latency and larger peak amplitude (Figure 3e). Previous studies showed that the human EC is functionally segmented along its anterior–posterior axis, in accordance with reported anatomical connectivity patterns of EC in rodents and nonhuman primates along its medial–lateral axis (Hafting, Fyhn, Molden, Moser, & Moser, 2005; Maass, Berron, Libby, Ranganath, & Duzel, 2015; Yarstev, Witter, & Ulanovsky, 2011). Furthermore, another human fMRI study showed that the anterior–posterior axis of the hippocampus and the EC provides a representation of a scaling of spatial relationships in a graded manner, most detailed in posterior areas (Evensmoen et al., 2015). Notably, the anterior–posterior gradient within the EC, as we found, provides direct evidence for the functional subdivisions along the anterior–posterior axis in the human EC.

Finally, the correlation between P1w peak amplitudes and WMS‐R score also suggests that P1w potentials may be potential biomarkers of memory impairment. In three patients, in whom we added the scalp electrode for the sleep staging in sleep CCEP, the P1w potentials were recorded in not only the intracranial subdural electrodes but also in the scalp electrodes (Figure S4). Grossman et al. (2017) proposed a novel noninvasive strategy for electrically stimulating neurons at depth via temporally interfering electric fields from the scalp electrodes. If the human EC could be stimulated noninvasively, the P1w potentials can be evoked and recorded noninvasively without intracranial electrode implantation. In the future, such a technical innovation may provide a new opportunity for the evaluation of clinical usefulness of scalp‐recorded P1w potentials as a memory impairment biomarker in various neurological diseases with memory dysfunction, such as Alzheimer's disease.

### 4.5 Limitations of the present study

There are several limitations in the present study. The number of subjects recruited for the study was relatively small (*n* = 9). According to the clinical purpose of the functional brain mapping, such as language function mapping, the majority of the patients (7 out of 9 patients) had the electrode coverage on the left hemisphere. The evoked responses to the electrical stimulation of the right EC were recorded in only two patients. Thus, it was difficult to confirm the characteristics of the responses to the right EC stimulation, or whether the unique CCEP responses shown in the present study were specific to the left EC stimulation.

As for the supposed generator mechanism of P1w, we provided only limited evidence supporting the hypothesis that P1w reflects a far‐field potential from the medial temporal structures. The majority of the implanted electrodes were subdural electrodes. Therefore, the activity in deep structures of the brain, such as the hippocampus or the thalamus, was not recorded in most patients. Thus, the confirmation of the P1w generator mechanism was difficult.

In the present study, we could not standardize the current intensity of the stimulation, which ranged between 6 mA and 12 mA, due to symptoms such as pain, excessive artifacts in adjacent electrodes, or afterdischarges. However, we considered that the difference of the stimulus intensity among the patients in the range between 6 and 12 mA may not invalidate our results. This would be supported by the results as follows: We observed only small differences as for the presence ratio, peak latency, and peak amplitude of P1w among 6‐, 8‐, and 12‐mA stimulation (presence ratio: 90.2% [6 mA], 87.0% [8 mA], 92.4% [12 mA]; peak latency: 11.2 ms [6 mA], 10.2 ms [8 mA], 10.4 ms [12 mA; in average]; peak amplitude: 32.1 μV [6 mA], 30.8 μV [8 mA], 35.0 μV [12 mA; in average]) in the additional analysis of CCEP responses to the stimulation of the same EC stimulus site with variable current intensity (1, 2, 4, 6, 8, 12 mA) in one patient (the patient G). However, further study with standardized stimulus parameters is needed to further validate the results in the present study.

Since all subjects suffer from medial temporal lobe epilepsy, we could not completely exclude the possibility that P1w is entirely a pathological response through aberrant epileptic networks involving the EC. However, two out of nine patients (patients C and E) did not have hippocampal sclerosis nor was the epileptic focus located in the parahippocampal gyrus (Table 1). This supports the hypothesis that P1w reflects the activity of a normal memory network.

## 5 CONCLUSIONS

We clarified that human EC electrical stimulation evoked short‐latency potentials on the broad neocortical regions. The features of those potentials, termed as P1w, were positive polarity, short peak latency (mean 20.1 ms), and waveform uniformity. The origin of P1w remains unclear, although the limited evidence from the present study suggests that P1w is the far‐field potential by the volume conduction of giant evoked potential from the medial temporal structures, especially the EC itself and hippocampus. A stimulation of more posterior parts of the EC induced P1w with shorter latency and larger amplitude. Our results support to a certain extent the idea that P1w reflects memory circuit activity, as suggested by the correlation between the WMS‐R score and the peak amplitude of P1w, although that correlation did not survive a correction of multiple comparisons. The significance of the present study is that those evoked potentials may be a potential biomarker of memory impairment in various neurological diseases, and we provided direct evidence for the functional subdivisions along the anterior–posterior axis in the human EC.

## CONFLICT OF INTEREST

Department of Epilepsy, Movement Disorders and Physiology is an endowment department, supported with a grant from GlaxoSmithKline K.K., NIHON KOHDEN CORPORATION, Otsuka Pharmaceutical Co., and UCB Japan Co., Ltd. Department of Respiratory Care and Sleep Control Medicine is the department donated from Teijin Pharma Limited, Philips Respironics GK, Fukuda Denshi CO., LTD., and Fukuda Lifetech Keiji CO., LTD.

## Data Availability

The data that support the findings of this study are available on request from the corresponding author. The data are not publicly available due to privacy or ethical restrictions.
