# Supplementary material for: Human entorhinal cortex electrical stimulation evoked short‐latency potentials in the broad neocortical regions: Evidence from cortico‐cortical evoked potential recordings
Source: Brain Behav. 2019 Jul 30;9(9):e01366. doi: 10.1002/brb3.1366 (PMC6749511; doi:10.1002/brb3.1366)
Supplement: Supplementary file 5 [file BRB3-9-e01366-s005.docx]

Supplementary Figure 1. N1 definition

Representative CCEPs evoked by EC stimulation in patient D. The arrowheads indicate N1 peaks. The area shaded in grey indicates the inclusion criteria for N1 as defined in Materials and Methods.

A) Typical N1, recorded from the inferior parietal area. A typical N1 shows a peak latency between +5 and +50 ms and is followed by N2.

B) Late N1. The peak latency is longer than that of typical N1. The amplitude should exceed six SD of the baseline activity (shaded in grey).

In both typical and late N1, the amplitude should exceed six SD of the baseline activity between +5 and +50 ms, either at the ascending slope or at the peak of the N1 potential. The waveforms included by amplitude criteria are shaded in grey between +5 and +50 ms.

Supplementary Figure 2. Location of local maxima in remote isolated fields.

Black dots show locations of electrodes that recorded local maxima of the remote isolated fields, presented in the MNI standard space across all patients. All locations are mapped to the left hemisphere for display purposes. In contrast to FG stimulation, local maxima of the remote isolated field in EC stimulation are almost limited to the temporal lobe and orbitofrontal area.

Supplementary Figure 3. Effects of the reference electrode on CCEP waveforms.

A, B) CCEPs evoked by EC stimulation in a representative case, patient F. CCEPs are shown for different reference electrodes: mastoid electrode located on the contralateral mastoid process (A) and the electrocardiogram electrode located in the supraclavicular area (extra-cranial region: B). CCEP waveforms in panel A and B are essentially the same. All conventions are taken from Figure 2.

C) Superimposed CCEP waveforms of lateral surface electrodes. Left and right panels show superimposed responses for mastoid and electrocardiogram reference electrodes, respectively. Only marginal differences were seen between the two superimpositions.

D) Averaged response at the mastoid electrode with reference to the electrocardiogram electrode. No clear evoked potentials were observed. Therefore, it is unlikely that P1w is merely a consequence of the reference electrode activation.

Supplementary Figure 4. P1w potentials recorded in the scalp electrodes.

The figures show P1w potentials recorded in the scalp electrodes and subdural electrodes, with reference to the electrocardiogram electrode, at the awake state during sleep CCEP in patient F (Right EC stimulation, 6 mA, 1 Hz). The right upper figure shows the superimposed waveforms recorded in the subdural electrodes on the lateral side of the right hemisphere. The scalp electrodes were placed according to the international 10-20 system. The peak latencies of those P1w potentials were almost identical at around 10 ms, suggesting the same origin.
